# New insights of Nb_2_O_5_-based coatings on the 316L SS surfaces: enhanced biological responses

**DOI:** 10.1007/s10856-021-06498-7

**Published:** 2021-03-06

**Authors:** Jéferson Aparecido Moreto, Rogério Valentim Gelamo, Marcos Vinicius da Silva, Teresa Tromm Steffen, Carlo José Freire de Oliveira, Patrícia Andressa de Almeida Buranello, Marcelo Rodrigues Pinto

**Affiliations:** 1 Institute of Exact Sciences, Naturals and Education, Federal University of Triângulo Mineiro (UFTM), Avenida Doutor Randolfo Borges Júnior, Univerdecidade, Uberaba, Minas Gerais Brazil; 2 Institute of Technological and Exact Sciences, Federal University of Triângulo Mineiro (UFTM), Avenida Doutor Randolfo Borges Júnior, Univerdecidade, Uberaba, Minas Gerais Brazil; 3 Laboratory of Immunology and Bioinformatics, Institute of Natural and Biological Sciences, Federal University of Triângulo Mineiro, Uberaba, Minas Gerais Brazil; 4 Center for Technological Sciences, UDESC, Joinville, Santa Catarina Brazil; 5 Department of Dentistry, University of Uberaba, Uberaba, Minas Gerais Brazil

## Abstract

This communication aims to propose new insights of Nb_2_O_5_-based coatings on the 316L SS surface with great prospects to be used in the dentistry field as brackets. The Nb_2_O_5_ thin film was incorporated into the 316L SS by using PVD method. For this purpose, the studied system was characterized structurally and morphologically by using AFM, FTIR-IRRAS, Raman spectroscopy and X-ray photoelectron spectroscopy (XPS). Biological assays were performed using human gingival fibroblast cell-line HGF-1. In agreement with FTIR and Raman results, the XPS technique indicates that Nb is present in an oxidation state assigned to Nb_2_O_5._ Furthermore, the coatings produced by PVD technique are less toxic and induces less inflammation in gingival cells (cell-line HGF-1), suggesting the strategy of use Nb_2_O_5_ thin film to cover the 316L SS promoted since its protection of the physiological environment to its biocompatibility improvement.

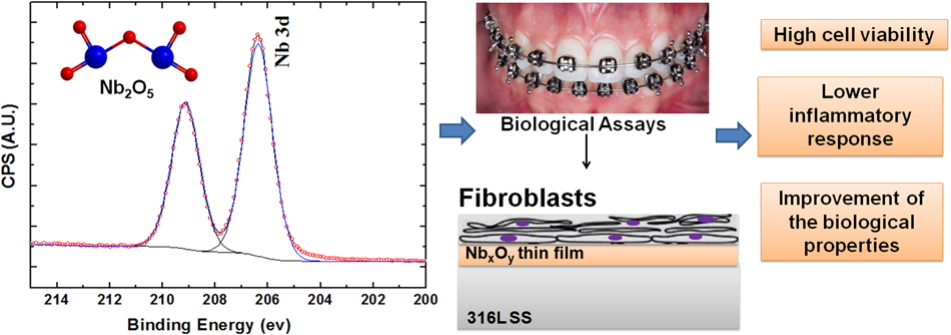

## Highlights

- PVD method was shown advantageous for producing Nb_2_O_5_ thin film on the 316L SS.
- 316L SS coated with Nb_2_O_5_ film to be used in biomedical devices.
- Nb_2_O_5_ coating improved biological response of the 316L SS.
- Nb_2_O_5_ thin film are less toxic and induces less inflammation in gingival cells.

## Introduction

The development of new biomaterials has been growing in recent decades and among the requirements for its use one can cite, good resistance to the wear process, good corrosion resistance in media containing aggressive ions and biocompatibility [1]. Many methods of surface treatments can be used to obtain biomaterials with a wide variety of applications, such as anodizing [2], chemical conversion coating [3], electrodeposition [4], plasma enhanced chemical vapor deposition [5] and physical vapor deposition (PVD) [6]. Although conventional materials such as 316L stainless steels (SS), titanium alloys containing hydroxyapatite doped with rare earth elements [7], titanium alloys coated with diamond-like carbon thin films [8], Co–Cr alloys and magnesium alloys have been used for a long time as biomaterial, there are still several limitations to their use [9]. Improving the biological responses of 316L SS is a long-standing and active area of biomedical research [10]. However, most studies on corrosion processes in aggressive media found in the literature do not associate them with biological effects such as cytotoxicity and biocompatibility. The biomaterials to be used in clinical practice need to fulfill many requirements once it needs to ensure the safety and effectiveness to the patient [10]. Among these requirements, we may mention the absence of cytotoxic activity, irritation and induction of the immune and inflammatory response by the biomaterial. Here, we provide new insights of Nb_2_O_5_-based coatings on the 316L SS surface, validating its applicability potential in dentistry field to be used as brackets.

## Material and methods

Specimens of 316L SS were ground with SiC abrasive paper from 600# to 4000#. Prior the deposition, the samples were immersed in neutral detergent during 1 h, rinsed with distilled water, dried and cleaned with 70% (v/v) alcohol. The Nb_2_O_5_ thin films were deposited using argon (99.99%) and oxygen (99.99%) at 5.0 and 0.5 mTorr in a DC-magnetron sputtering [11]. Over a Niobium (99.99%) 2-in. diameter target was applied 440 V and 140 mA resulting film of 300 nm thick.

The topography of the films was investigated by using AFM (Shimadzu SPM 9700 Microscope equipment) in tapping mode. The structural characterization of the 316L SS containing Nb_2_O_5_ coatings was performed by FTIR technique. The spectra were collected 64 scan s^−1^ using an Agilent Cary 640 spectrometer in the range of 4000–500 cm^−1^. An IRRAS (Infrared reflection-absorption spectroscopy) apparatus from Pike Veemax incidence at 70° was used. Raman spectroscopy was performed with a Horiba LabRAM HR Evolution instrument equipped with a confocal microscope. Spectra were collected using a 532 nm Ar^+^ laser source, 50 mW power, 100× objective in the range 80–4000 cm^−1^. The thin film was also characterized by X-ray Photoelectron Spectroscopy (XPS), using a K-Alpha Thermo Scientific spectrometer (X-ray Al-Kα, *hν* = 1486.6 eV), at a pressure below 10^−7^ mBar. Pass energy applied to obtain the spectra was 200 eV for survey and 50 eV for high-resolution scans. The spectra deconvolution was performed using the Voigtian function, with Gaussian (70%) and Lorentzian (30%) combinations.

Biological assays were performed using human gingival fibroblast cell-line HGF-1. Gingival fibroblasts HGF-1 (CRL-2014, ATCC) were cultured in T-25 culture vessels (Nunc), temperature of 37 °C, in an atmosphere of 5% CO_2_, with Dulbecco’s Modified Eagle’s Medium (DMEM) enriched with 10% FBS (Sigma-Aldrich, USA). DMEM medium was renewal 2 to 3 times per week until HGF-1 cells reach an 80–90% confluence. After, cells were removed using 0.25% trypsin, 0.53 mM EDTA solution and seeded at 1 × 10^5^ cells/mL on the uncoated and coated 316L SS with Nb_2_O_5_ thin film. After 24 h of incubation at 37 °C, 5% CO_2_, cells were mechanically removed and evaluated for viability by flow cytometry using Annexin-V plus 7-ADD staining, performed as manufacturer’s instructions (BD Biosciences, USA). Cell supernatants were used for quantification of TNF-α, IL-10, IL-17, IL-6, IFN-γ, and IL-4 cytokines by immunoenzymatic assay—ELISA, performed as manufacturer’s instructions (BD Biosciences, USA).

## Results and discussion

**Fig. 1:**
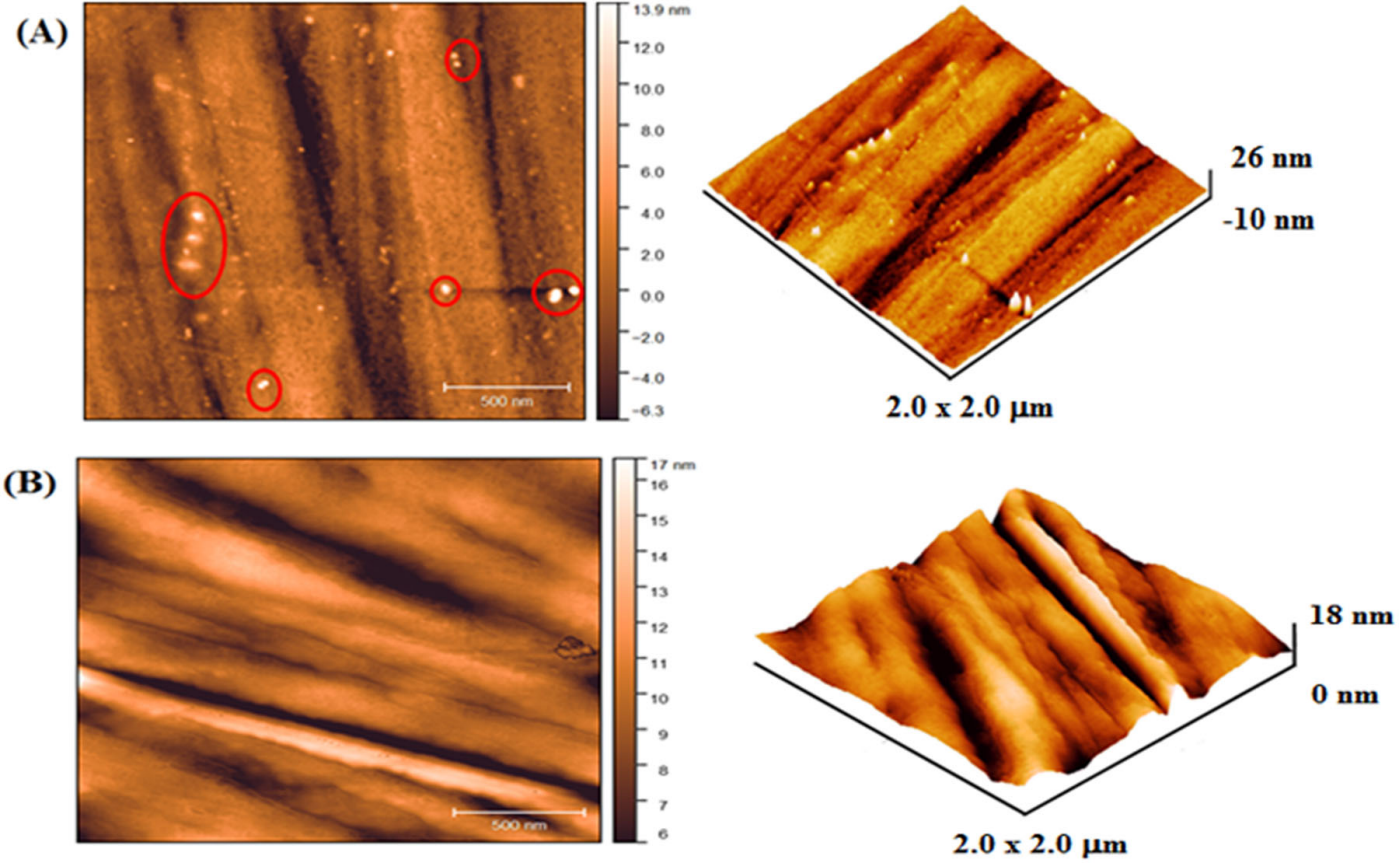
2D and 3D images of **A** 316L SS substrate, scale 2.0 × 2.0 μm and **B** 316L SS containing the Nb_2_O_5_ thin film, scale 3.0 × 3.0 μm

The topography of 316L SS coated and uncoated obtained by AFM are displayed at Fig. 1 and presents 2D and 3D images of the specimens studied here. The second-phase particles from the steel making process, which was marked with red circles are presented in Fig. 1A. The nucleation process in the vapor phase promoted the formation of a thin film that accompanies the risks arising from the sanding process (see Fig. 1B). The root means square surface roughness values obtained were 2.94 and 2.40 nm for the coated and uncoated 316L SS, respectively.

**Fig. 2:**
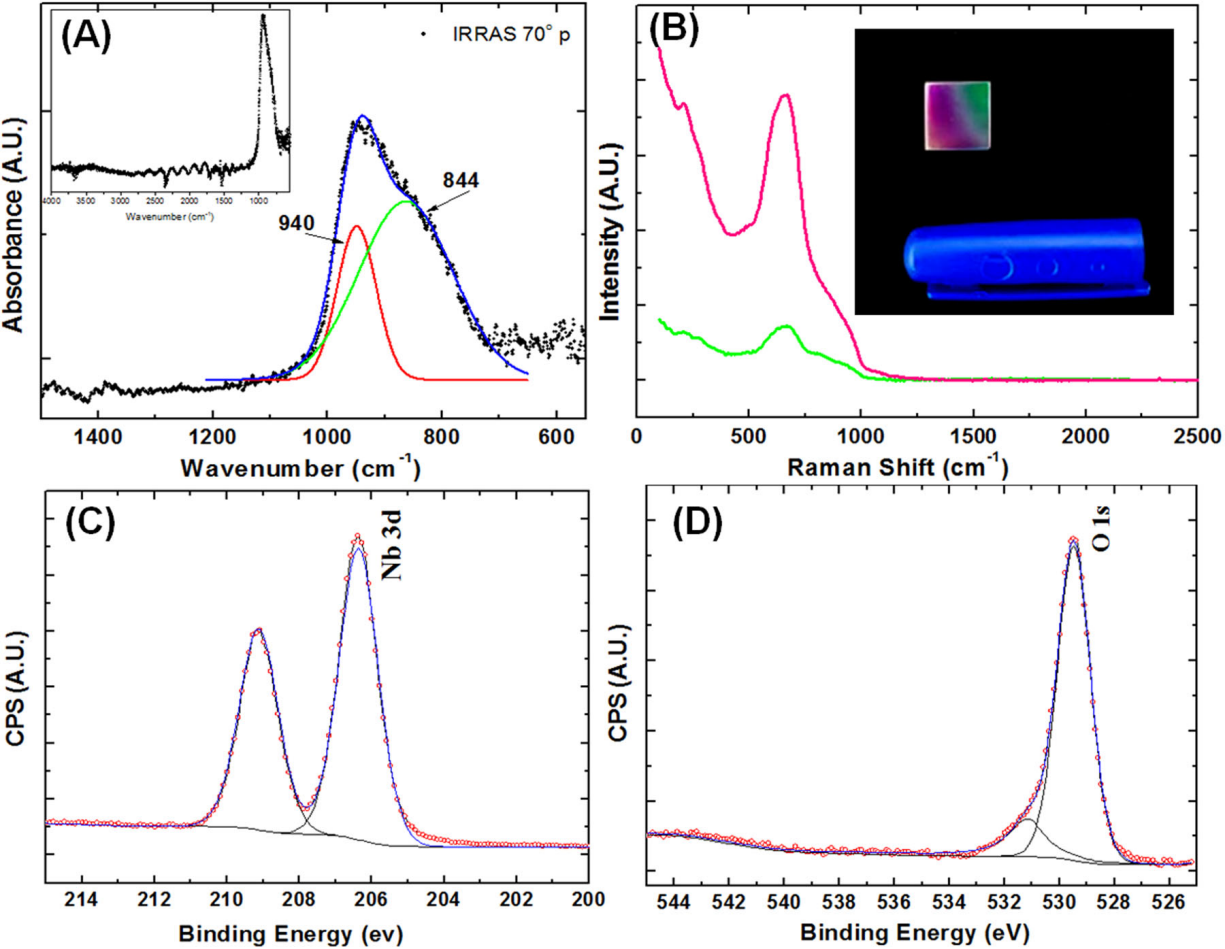
**A** FTIR-IRRAS spectrum obtained at 70° p-polarized for Nb_2_O_5_ thin film showing the bands centered at 844 and 940 cm^−1^ (inset displays the FTIR spectra in the range 4000 to 500 cm^−1^). **B** Raman spectra for the 316L SS containing Nb_2_O_5_ film (inset displays the different regions of the film and its spectra). **C** Nb 3*d* levels and **D** O 1*s* XPS deconvolution spectra

FTIR spectra of the Nb_2_O_5_ thin film in range of 1400–600 cm^−1^ is presented in Fig. 2A. The spectrum resulted in an intense band between 1100 and 700 cm^−1^. The deconvolution of this large band resulted in two other bands centered at ~940 and ~844 cm^−1^ attributed to Nb=O and Nb–O–Nb stretching [12, 13], respectively. The inset in Fig. 2A displays the whole IR spectra of Nb_2_O_5_ film herein studied indicating no additional bands related to the niobium oxide film. The major Raman band appears at ~655 cm^−1^ as well as another band presenting a lower intensity around 206 cm^−1^ attributed to Nb_2_O_5_ [14] (see Fig. 2B).

Survey XPS indicates the film chemical composition as 53 at.% of oxygen, 27 at.% of carbon and 20 at.% of niobium. The chemical states of niobium and oxygen were determined by high-resolution scans deconvolution, showed in Fig. 2C, D. Nb 3*d* deconvolution shows doublets for Nb 3*d*_5/2_ and Nb 3*d*_3/2_, at 206.4 eV and 209.1 eV, respectively. In agreement with FTIR and Raman results, it indicates that Nb is present in an oxidation state assigned to Nb_2_O_5_ [15, 16]. Also, O 1*s* deconvolution presents a major contribution (83 at.%) of the peak at 529.5 eV, assigned to Nb_2_O_5_ [16]. The other peak, at 531.1 eV, is related to –OH groups and/or oxidized carbon species [17]. These results confirm the thin film formation preferentially composed by niobium in Nb_2_O_5_ oxidation state.

**Fig. 3:**
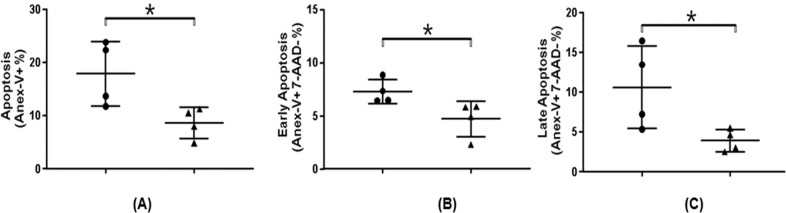
HGF-1 cell-line cultured on the coated and uncoated 316L SS substrates, **A** total, **B** early, and **C** late apoptosis. Points represent each independent culture and lines the mean ± standard deviation. *P < 0.05. (●) 316L SS and (▲) 316L SS coated Nb_2_O_5_

The biological results presented that the 316L SS containing the Nb_2_O_5_ thin film induces significantly less cell death, as demonstrated by total apoptosis (Annexin-V + cells, *p* = 0.02), early apoptosis (Annexin-V + 7-ADD—cells, *p* = 0.02) and late apoptosis (Annexin-V + 7-ADD + cells, *p* = 0.04) evaluation. Figure 3 displays the HGF-1 cell-line cultured on the coated and uncoated 316L SS substrates. As can be seen, the total (A), early (B) and late apoptosis (C) are demonstrated. The results showed the Nb_2_O_5_ thin film improved the biocompatibility of the 316L SS substrate. Probably, the increase of the biocompatibility may be related to the surface properties, particularly the surface energy and surface wettability as related by Hao and Lawrence [18]. Niobium coatings are biocompatible to fibroblast cells that assumed the elongated fibroblastic appearance typical of osteoblast-like cells. The results obtained in the present work are in accordance to Eisenbarth et al. [19], which attributes the increase viability, proliferation, mitochondrial and osteogenic activity of titanium implants to the niobium-based films on the substrate surfaces.

**Fig. 4:**
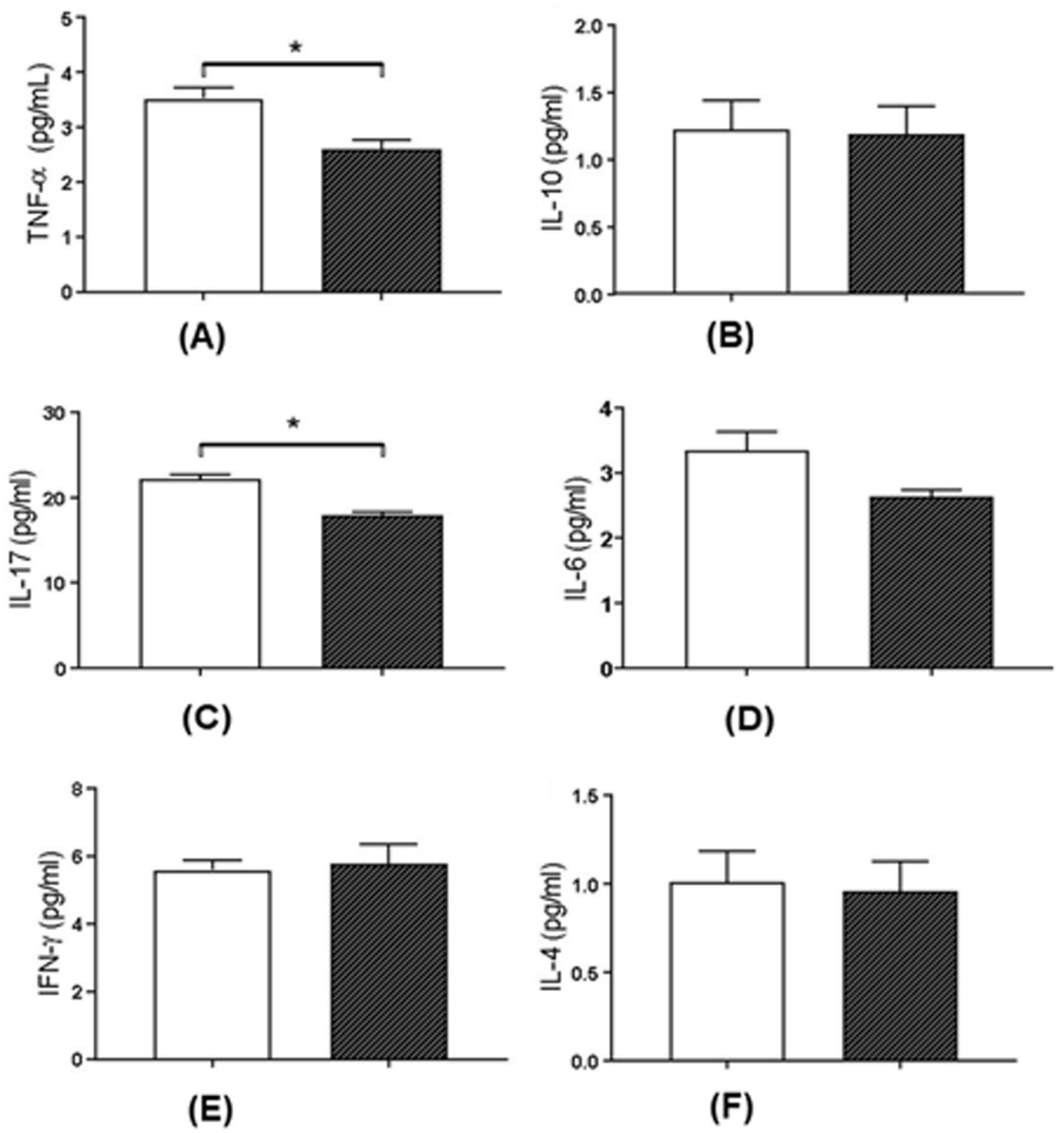
Effect of 316L SS coated and uncoated in cytokine production of HGF-1. The cells were cultured for 24 h on both specimens. After 24 h of culture supernatant was collected and TNF-α (**A**), IL-10 (**B**), IL-17 (**C**), IL-6 (**D**), IFN-γ (**E**) and IL-4 (**F**) were analyzed by cytometric bead array (CBA). Bars represent the mean ± standard deviation of cytokine production from quadruplicate samples. *P < 0.05. (▭) 316 L SS and (▬) 316L SS coated Nb_2_O_5_

In addition to improving cell compatibility by reducing cell toxicity of tested surfaces, it is desirable that biomaterials do not induce harmful inflammation after cell contact. In this context, the 316L SS containing the Nb_2_O_5_ thin film induces significantly less TNF-α production, a huge pro-inflammatory cytokine involved in a diversity of inflammatory and autoimmune diseases (*p* = 0.02), and similar levels of IL-10, a classical anti-inflammatory cytokine (see Fig. 4A, B). Besides, the 316L SS containing Nb_2_O_5_ film induced less production of IL-17 (*p* = 0.001), other cytokine deeply involved in inducing and mediating pro-inflammatory responses (Fig. 4C). We also observed a similar production of IL-6, besides a trend to lower levels in 316L SS coated with Nb_2_O_5_ (*p* = 0.06). Like TNF-α and IL-17, IFN-y, and IL-4 are important inflammatory cytokines, however, these molecules were not changed in the culture with all the films tested (Fig. 4D–F).

TNF-α is directly involved in inflammation maintenance and activation of osteoblasts, inducing bone desorption, and films that act inducing lower concentrations are more biocompatible in a long term [20], such as demonstrated by lower levels of TNF-α production by murine macrophage cells cultured with niobium-covered surfaces [21]. In a similar way, IL-17 is a huge pro-inflammatory cytokine associated with chronic inflammation [22], osteoclastogenesis [22] and that can amplify inflammatory responses by inducing TNF-α secretion by macrophages [23].

## Conclusion

The PVD method was shown advantageous for producing Nb_2_O_5_ thin film on the 316L SS surface. The coatings produced are less toxic and induces less inflammation than 316L SS in gingival cells (cell-line HGF-1) with high prospects to be used in the dentistry field as orthodontic brackets. The biological results suggest that the strategy of use Nb_2_O_5_ thin film to cover the 316L SS promoted since its protection of the physiological environment to its biocompatibility improvement. Furthermore, this study is the starting point for the development of robust studies in biomedical area by using Nb_2_O_5_ thin film as a protective barrier against corrosion process and enhanced biological responses.
